# Cultivation-independent approach for the direct detection of bacteria in human clinical specimens as a tool for analysing culture-negative samples: a prospective study

**DOI:** 10.1186/s40064-016-1949-3

**Published:** 2016-03-15

**Authors:** Ma. Guadalupe Aguilera-Arreola, Marcos Daniel Martínez-Peña, Fabiola Hernández-Martínez, Sara R. Juárez Enriques, Beatriz Rico Verdín, Cristina Majalca-Martínez, Graciela Castro-Escarpulli, Enrique Albarrán-Fernández, S. Cecilia Serrano-López

**Affiliations:** Department of Microbiology, Instituto Politécnico Nacional (IPN), Escuela Nacional de Ciencias Biológicas (ENCB), Mexico City, D.F. Mexico; Special Test Laboratory, Centro Médico Nacional (CMN) 20 de Noviembre - Instituto de Seguridad y Servicios Sociales de los Trabajadores del Estado (ISSSTE), Mexico City, D.F. Mexico; Department of Epidemiology, Centro Médico Nacional (CMN) 20 de Noviembre del Instituto de Seguridad y Servicios Sociales de los Trabajadores del Estado (ISSSTE), Mexico City, D.F. Mexico; Microbial Genetic Resources Laboratory, Centro Nacional de Recursos Genéticos (CNRG)-INIFAP, Tepatitlán de Morelos, Jalisco Mexico; Department of Zoology, Escuela Nacional de Ciencias Biológicas - Instituto Politécnico Nacional, Mexico City, D.F. Mexico

**Keywords:** Hospital-acquired infections, Broad-range PCR, *16S rDNA*, Direct sequencing, Culture-negative

## Abstract

Administration of empirical antibiotic therapy prior to microbiological diagnosis is thought to be associated the failure of subsequent bacterial growth in culture. The aim of this study was to detect bacterial pathogens via direct amplification and sequencing of the *16S rDNA* gene in samples showing negative culture results as alternative diagnostic tools to troubleshoot difficult samples. Twenty-three (7.66 %) positive samples were detected, most of which were monomicrobial infections; 15 of the cases were identified as HAIs, 6 had catheter colonisation, and 2 had sample colonisation. The pathogens identified included *Escherichia*, *Salmonella*, *Pseudomonas* spp., *Enterococcus* spp. and coagulase-negative staphylococci (CoNS). The most frequent infections were bacteraemia and urinary tract infection, but meningitis, warm infection and soft tissue infection were also documented. These findings emphasise the efficacy and usefulness of molecular diagnosis, thus *16S rDNA* gene analysis is strongly indicated by HAIs diagnostics.

**Electronic supplementary material:**

The online version of this article (doi:10.1186/s40064-016-1949-3) contains supplementary material, which is available to authorized users.

## Background

Over the last two decades, broad-range polymerase chain reaction (PCR) analysis of bacterial *16S rDNA* genes has proven to be a useful tool, not only to establish phylogenetic bacterial relationships but also to identify uncharacterised bacterial isolates, as well as bacterial pathogens, directly from clinical specimens (Petti 2007). Bacterial *16S rDNA* genes generally contain nine “hypervariable regions” that demonstrate considerable sequence diversity among different bacterial species. These regions are flanked by conserved stretches in most bacteria, enabling PCR amplification of target sequences using universal primers (Chakravorty et al. 2007). Diverse sets of broad-range PCR primers directed against conserved regions of the *16S rDNA* gene have been designed to specifically amplify several bacterial genera (e.g., the V1-V3 region) (Chakravorty et al. 2007; Clarridge III 2004; Nikkari et al. 2002); universal primers are typically designed to be complementary to the conserved regions located at the beginning of genes (approximately 540 bp). The resulting amplified 16S *rDNA* sequences allow for adequate differentiation of bacteria with a sufficiently high rate of detection, thereby establishing a reliable basis for in silico analyses for identification by *16S rDNA* gene sequencing (Clarridge 2004). Thus, investigation of clinical samples using this methodology represents a cultivation-independent alternative approach for the detection of nonviable bacteria and bacteria with fastidious growth requirements in various clinical samples.

Healthcare-associated infections (HCAIs) result in mortality, morbidity, and increased healthcare costs worldwide (Labelle et al. 2010). Microbiological identification of the causative organism is well recognised to be crucial in the treatment of HCAIs (Saito et al. 2012). Some reports have indicated that the administration of empirical antibiotic therapy prior to microbiological diagnosis is associated with the failure of subsequent bacterial growth in culture. Additionally, negative culture results in the microbiological diagnosis of hospital-acquired infections (HAIs) have been attributed to several factors, including inadequate sample collection and the time elapsed between sample collection and analysis (Nikkari et al. 2002; Bhattacharya and Mondal 2010). In these particular cases, molecular diagnosis can be quite useful. For example, this approach has been particularly valuable for the diagnoses of brain abscess, aortic infection, bacteraemia, pneumonia and liver abscess and prosthetic joint infections (Kommedal et al. 2009, Labelle et al. 2010; Saito et al. 2012; Hartley and Harris 2014). The aim of this paper was to perform a preliminary study to determine whether this approach of broad-range bacterial *16S rDNA* PCR and direct sequencing is particularly valuable for implementation and to identify key diagnostic findings to improve the diagnosis of infections in hospital laboratories.

## Methods

### Sample collection and ethical statement

Over a period of 3 months, 300 culture-negative specimens from different anatomic sites were collected at the Laboratory of Special Tests at the National Medical Centre (Centro Médico Nacional (CMN) 20 de Noviembre) of the Institute of Security and Social Services for State Workers (Instituto de Seguridad y Servicios Sociales de los Trabajadores del Estado, ISSSTE) in Mexico City. Once the clinical laboratory reported that samples were negative by culture (and before properly discarding the samples as biowaste), 1 mL of each sample was placed in a 1.5-mL tube for DNA extraction for use in the present study. In accordance with all applicable federal regulations concerning the protection of human subjects (General Health Low, Title Two, Articles 17 and 23), acquiring informed consent from patients is not mandatory for safe research (when no risk is imposed upon the patients). This study was classified as safe research because all cultures were ordered by physicians due to the necessity of clinical management, and any samples specifically collected for this study, in addition to the datasets, were de-identified. The ethics committee “CMN 20 de Noviembre” reviewed and approved the protocol (number 014-2012).

### Negative culture sample definition

Samples were included in the present study presenting any evidence of bacterial infection other than that observed by clinical examination. Samples were deemed culture negative if any aetiologic agents were recovered for 7 days at 37 °C using the standard microbiology laboratory procedures. The samples were analysed for the presence of bacteria and fungi.

### DNA extraction from specimens

DNA extraction was performed using a High Pure PCR Template Preparation Kit (Roche Diagnostics, Indianapolis, IN) according to the manufacturer’s instructions. Briefly, 200 µL of sample was incubated at 76 °C with Binding Buffer provided by the kit and proteinase K, followed by washes with isopropanol, an inhibitor removal solution and washing buffer. Finally, the DNA was eluted and stored at −20 °C until use.

## *16S rDNA* amplification

Broad-range PCR targeting the *16S rDNA* gene V1-V3 variable regions was performed as previously described (Kommedal et al. 2009), with certain modifications as stated below. PCR amplification was performed in a final volume of 25 µL, containing the DNA sample, 1X PCR buffer (Invitrogen™, Carlsbad, CA), 2.5 U *Taq* DNA recombinant polymerase (Invitrogen™, Carlsbad, CA), 0.25 mM deoxynucleotide triphosphate (dNTP) mix (Invitrogen™, Carlsbad, CA), 0.1 µM of each primer (forward primer 5′ TTG-GAG-AGT-TTG-ATC-MTG-GCT-C 3′ and reverse primer 5′ GTA-TTA-CCG-CGG-CTG-CTG 3′) and 1 mM MgCl_2_; 0.6 % bovine serum albumin (BSA) was added to prevent PCR inhibition. All reactions were performed with a T-Gradient Thermoblock PCR System (Biometra, Goettingen, Germany). The cycling conditions used to amplify the ~510 bp product consisted of initial denaturation at 94 °C for 5 min, 30 cycles of melting at 94 °C for 30 s, annealing at 53.2 °C for 1 min, and elongation 72 °C for 1 min, and a final extension step at 72 °C for 5 min. To verify the presence of the amplification products, 5 µL of each PCR-amplified sample was separated by electrophoresis on a 1.0 % agarose gel. A 100-bp DNA molecular marker was included in some of the electrophoresis runs (Invitrogen™, Carlsbad, CA). The remaining 20 µL of sample was frozen for later use. Each sample was run in parallel with a negative control (with addition of distilled water instead of DNA) and a positive control (with addition of DNA from a positive sample that had been previously tested).

### Sequencing and identification

A phylogenetic tree was constructed to show the relationships of the sequences identified by sequence analysis. The PCR products were cleaned using a Montage Gel Extraction Kit (Concord Road, Billerica, MA) according to the manufacturer’s instructions and were authenticated via DNA sequencing with an ABI-PRISM™ 310 system (Applied Biosystems, Foster City, CA) according to the manufacturer’s recommendations.

**Fig. 1:**
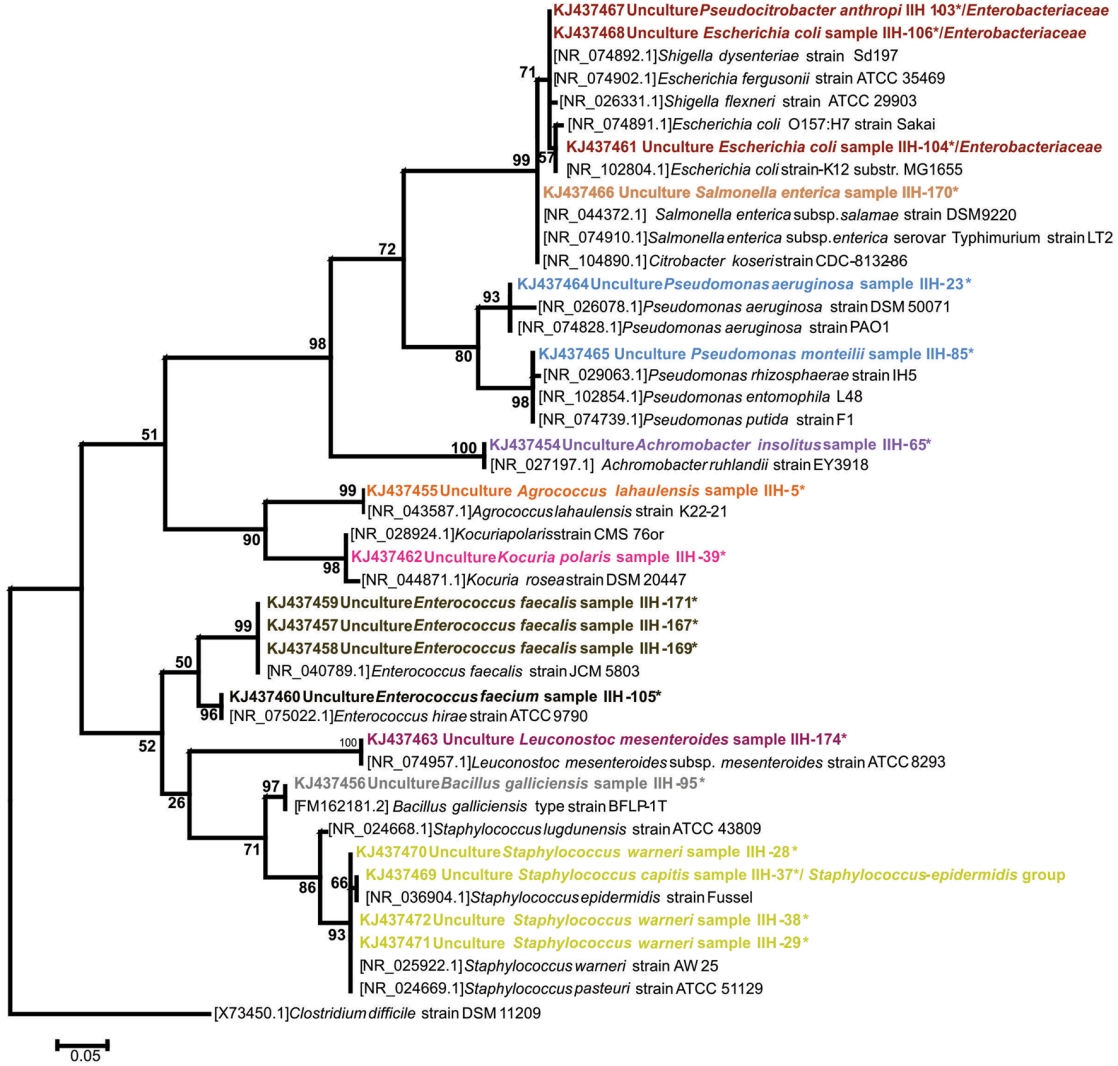
Bacteria identification to the species level by broad-range *16S rDNA* PCR and sequencing from 19 samples with negative culture. This general tree was generated using the neighbour-joining (NJ) with the Tamura–Nei model. The *numbers* shown at the branch point indicate bootstrap values. The dataset was subjected to 1000 bootstrap replications. The sequences were selected from the GenBank database, and their accession numbers are listed in *brackets*. Sequences with an *asterisk* correspond with the clinical samples from this study, and the sequence accession numbers are GenBank KJ437454 to KJ437472. All the sequences could be an assign unambiguously to a genus. The species designation was determining with the closest genetic identification based on GenBank sequence analysis

Initially, DNA sequences amplified using the forward and reverse primers were assembled. The obtained sequences (n = 19) were edited to exclude the PCR primer-binding sites, and ambiguous and incorrectly called bases were manually corrected using BioEdit version 7.0.9.0 (Hall 1999), CLUSTAL_X (Larkin et al. 2007) and Seaview version 4.3.3 (Gouy et al. 2010). All sequences were compared to those available for the V1-V3 regions of the *16S rDNA* gene in GenBank DNA databases (nr/nt and refseq_rna) using the BLAST algorithm (www.ncbi.nih.gov) (Morgulis et al. 2008). Multiple sequence alignments were performed using Clustal X version 2.0 (Larkin et al. 2007), and the alignments were edited with SeaView (Galtier et al. 1996). The analyses were performed using the default setting parameters in all the software’s. The identities of the sequences of genus and species was presumed to be correct for clinical samples if sequence identities was of 97 and 99 %, respectively to reference sequences of strains match in GenBank and EZTaxon databases (Kim et al. 2012; Morgulis et al. 2008; Stackebrandt and Ebers 2006; Rosselló-Mora and Amann 2001). A phylogenetic tree was constructed for each sequence using the maximum parsimony method (MP) and neighbour-joining (NJ), in the NJ method the substitution model of Tamura–Nei was used. Phylogenetic analyses were conducted using Mega 6 (Tamura and Nei 1993; Tamura et al. 2013). The stability or accuracy of each inferred topology was assessed by bootstrap analysis with 1000 replicates (data not shown). Finally, using the consensus sequences of assigned identification, a general NJ tree was constructed using the substitution model of Tamura–Nei and Mega 6 software (Tamura and Nei 1993; Tamura et al. 2013). The stability or accuracy of each inferred topology was assessed by bootstrap analysis with 1000 replicates. The sequences included were intentionally selected from the GenBank database in order to highlight the species identification. Their accession numbers are listed in brackets (Fig. 1).

Mixed DNA chromatograms were analysed using the RipSeq web application (iSentio). The RipSeq mixed algorithm searches against the “16S human pathogen iSentio” database (Kommedal et al. 2009). The sequences obtained herein were submitted to the GenBank database under the accession numbers KJ437454 to KJ437472.

### Epidemiological information

To confirm the HAIs, the official Mexican standard NOM-045-SSA2-2005 criteria were used. The main clinical criteria for defining an HAI include fever, positive urinary culture, positive chest X-ray radiography results, symptoms observed on physical examination by a pulmonologist, positive phlegm and blood cultures, leucocytosis detection and evidence of an HAI in other bodily discharges. The data were collected using a checklist by the observer of the sample collection and were recorded in a series of clinical data documents. The patient microbiological information was collected and checked with respect to the date of admission, date of specimen culture, and culture results. The patients were visited by an infectious disease specialist, and all clinical manifestations of the patients were noted in the checklist. Initially, all 300 samples were considered positive for HAIs because they were obtained from patients who had been hospitalised for at least 48 h, and they were all collected because of clinically suspected infections.

## Results

This study included 300 culture-negative samples. Broad-range *16S rDNA* PCR showed positive results for 23 of these samples, demonstrating the presence of bacterial DNA. The relevant characteristics of the 23 positive cases are listed in Table 1. The sequences of 19 of the 23 amplicons were identified by rDNA analysis; 11 different genera were detected and identified using this methodology. Four mixed infections were detected by PCR, although identification using RipSeq Mixed software was not performed due to the low quality of the sequencing chromatograms. After clinical history revision, 15 of the 23 cases were defined as HAIs. Among the other 8 cases, 6 and 2 were classified as catheter colonisation and sample contamination, respectively (Table 1).

**Table 1:** Relevant characteristics of the positive broad-range PCR samples

Case number	Sample type	*16S* *rRNA* identification^a^	Hospital area	HAI	Symptomatology of HAI	Outcome
002-001	Bronchial aspirate	Mixed infection^b^	ICU-A	Undemonstrated bacteraemia	Yes	Improvement
002-005	CSF	*Agrococcus* sp.	ICU-N	Meningitis	Yes	UD
002-011	Blood culture	Mixed infection^b^	BU	Undemonstrated bacteraemia	Yes	Improvement
002-085	Blood culture	*Pseudomonas* sp.	Haematology	Bacteraemia	Yes	Death
002-088	Blood culture	Mixed infection^b^	Haematology	Sample contamination	Yes	Death
002-095	Blood culture	*Bacillus* sp.	Haematology	Bacteraemia	Yes	UD
002-114	Blood culture	Mixed infection^b^	Haematology	Bacteraemia	Yes	UD
002-174	Blood culture	*Leuconostoc* sp.	Haematology	Sample contamination	Yes	Improvement
002-028	Catheter tip	*Staphylococcus* sp.	BU	Catheter colonisation	Yes	Improvement
002-029	Catheter tip	*Staphylococcus* sp.	Cardiology	Catheter colonisation	Yes	Improvement
002-038	Catheter tip	*Staphylococcus* sp.	Cardiology	Catheter colonisation	Yes	Death
002-039	Catheter tip	*Kocuria* sp.	Haematology	Catheter colonisation	Yes	Improvement
002-167	Catheter tip	*Enterococcus* sp.	PIM	Catheter colonisation	Yes	Transfer
002-169	Catheter tip	*Enterococcus* sp.	Haematology	Bacteraemia	Yes	UD
002-023	Urine	*Pseudomonas* sp.	ICU-A	UTI	Yes	Death
002-065	Urine	*Achromobacter* sp.	Haematology	UTI	Yes	Death
002-103	Urine	*Pseudocitrobacter* sp.	ICU-A	Catheter colonisation	Yes	Death
002-104	Urine	*Escherichia* sp.	ICU-A	UTI	Yes	Improvement
002-105	Urine	*Enterococcus* sp.	Neurology	UTI	Yes	Transfer
002-106	Urine	*Escherichia* sp.	MMF	UTI	Yes	UD
002-170	Ulcer	*Salmonella* sp.	PI	Wound	Yes	UD
002-037	Wound secretion	*Staphylococcus* sp.	UTIP	STI	Yes	Death
002-171	Wound secretion	*Enterococcus* sp.	Rheumatology	Wound	Yes	Transfer

*CSF* cerebrospinal fluid; *ICU-A* intensive care unit, adult; *ICU-N* intensive care unit, new-born; *BU* burn unit; *PIM* paediatric internal medicine; *MMF* maternal foetal medicine; *ICU-P* intensive care unit, paediatric; *UTI* urinary tract infection; *PI* paediatric infectology; *STI* soft tissue infection; *HCAI* clinical healthcare infection; *UD* the outcome was not known at the time of the study

^a^The species designation was determining with the closest genetic identification based on GenBank sequence analysis (Fig. 1)

^b^Aetiological agents of polybacterial infections could not be identified due to low-quality sequences

Sufficient clinical evidence of an HAI and recognised infectious pathogens were detected in 9 of the 15 HAIs, including *Pseudomonas* (n = 2), *Enterococcus* (n = 3), *Escherichia* (n = 2), *Staphylococcus* (n = 1) and *Salmonella* (n = 1). The less common agents were *Agrococcus* sp., *Bacillus* sp. and *Achromobacter* sp. were also detected (3 cases). Mixed infection was detected in the other 3 cases, for which the aetiological agent could not be identified due to low sequence quality.

No evidence of infection was found in the medical records in the samples were coagulase-negative staphylococci (CoNS) (n = 3), *Kocuria* (n = 1), *Leuconostoc* (n = 1) and *Enterococcus* (n = 1) as tip or catheter colonisers. *Leuconostoc* was identified, probably as a contaminant of that particular sample as a result of poor sample management.

Although bacteraemia and urinary tract infection (UTI) were the most frequent infections, meningitis, warm infection and soft tissue infection were also documented.

Bacteria identified to the species level by broad-range *16S rDNA* PCR and sequencing from 19 samples with negative culture is show in the Fig. 1.

## Discussion

The accurate identification of bacterial isolates is one of the most important goals of a microbiology laboratory; such identification allows for the establishment of an effective antibiotic therapy as well as procedures that must be implemented to control the spreading of infection. Nevertheless, in the case of HCAIs, this step can be complicated due to several factors that affect culture sensitivity, such as adequate sample collection, the type of bacteria to be cultivated, and most importantly, the use of broad-spectrum antibiotics prior to sample collection (Nikkari et al. 2002; Bhattacharya and Mondal 2010; Rampini et al. 2011).

Due to the limitations of culturing, molecular techniques have been implemented in recent years for the detection and identification of bacterial pathogens. Gene amplification followed by sequencing is a promising diagnostic method, not only for pathogen identification but also for the discovery of unknown or “difficult to grow” microorganisms (Harris and Hartley 2003; Petti 2007). In our study, sixteen of the 23 positive patients had received broad-range antibiotic therapy with single or multiple antibiotics for long durations before the samples were collected, which could explain why the samples identified as positive by direct amplification and sequencing were culture negative (Additional file 1: Table S1).

Clinicians are frequently challenged by the suspicion of acute infectious disease, even when a conventional microbiological culture remains negative (Nikkari et al. 2002; Kommedal et al. 2009; Rampini et al. 2011). In the present study, this issue occurred in fifteen samples from patients clinically diagnosed with an HAI who had negative culture results. Overall, direct broad-range PCR amplification and sequencing, which can be used to identify recognised aetiological HAI agents, is a suitable supplemental method for the diagnosis of infections (Hartley and Harris 2014).

A retrospective medical record review showed that many samples submitted for microbiological analysis were obtained from patients without definitive evidence of an HAI. This issue has been previously reported by Rampini et al. (2011) and has been attributed to the difficulty of accurately diagnosing infections at the bedside. However, when laboratory findings (from 16S gene analyses) were considered in addition to clinical examination results in this study, at least 2 samples that were not previously clinically identified as HAIs were reclassified as HAIs, 2 additional samples were considered to contain contaminants, and 6 were considered to have catheter or tip colonisation. This finding highlights the utility of culture-independent identification as a complementary method for troubleshooting clinical specimens to support a clinical diagnosis. A quantitative study should be performed in the future because in almost all samples, except for those with catheter tip colonisation, the microbial load was critical for the clinical diagnosis of infections originating from the bacteria identified by molecular analysis. Among the analysed samples, the proposed analysis could be interesting for the testing of bronchial aspirates an additional diagnostic method; however, in some of the samples, the positive result was almost certainly due to a contaminant or pathogen with a non-significant bacterial load.

Some of the detected bacteria (CoNS and *Kocuria* sp.) are part of the normal skin microbiota. It is important to determine whether the isolation of these bacteria represents true infection or colonisation. The latter is quite common during specimen collection in patients with a long hospital stay. CoNS have emerged as predominant pathogens in HAIs and are recognised as the most common causes of bloodstream infection (BSI). Infection with CoNS is usually related to the use of intravascular devices, such as central venous catheters, peripheral venous catheters, haemodialysis catheters, and prosthetic materials. In this study, 4 cases of CoNS infection were found, 3 and one of which were associated with catheter tip colonisation and wound infection, respectively. In three cases, *Staphylococcus* closest to *S. warneri* was identified as a coloniser, and in one case, *Staphylococcus* closest to *S: capitis* was identified. Although this particular case was not considered an HAI because the patient did not present with signs of infection and given that *Kocuria* has been related to catheter-associated BSIs, the opportune detection of catheter colonisation by this bacterium is important for preventing the development of bacteraemia. The clinical significance of species other than *S. epidermidis* has been increasingly recognised in recent years; however, these species are rarely isolated. Nevertheless, *S. capitis* was detected in a wound secretion sample from a patient with an HAI. Given the ability of *Staphylococcus* closest to *S. capitis* to establish wound infections (associated biofilm-embedded bacteria) and because all clinical criteria were present in the infected patient, it is clear that this species was an aetiological agent of the infection, thereby indicating an HAI (Senger et al. 2007). On the other hand, *Staphylococcus* closest to *S. warneri* was isolated from catheter tips of patients with underlying immunosuppressive conditions, although peripheral blood cultures were not positive; in these cases, this bacterium was considered as having colonised the catheter or tip.

*Kocuria* sp. was detected in a 45-year-old male with acute lymphocytic leukaemia after 3 months evolution of the disease and who had been hospitalised for 3 days at the Haematology Service. Some *Kocuria* species have been associated with different HCAIs, including abscess and catheter-associated blood stream infection, and some authors have claimed that it is imperative to perform genetic analyses of *Kocuria* species because they are emerging pathogens (Lai et al. 2011; Tsai et al. 2010). Although this particular case was not considered an HAI because the patient did not present with signs of infection and given that *Kocuria* has been related to catheter-associated BSIs, the opportune detection of catheter colonisation by this bacterium is important for preventing the development of bacteraemia.

To date, *Agrococcus lahaulensis* and *B. galliciensis* have been described as environmental inhabitants, and there are no previous reports of their possible roles as aetiological agents of HAIs. In this study, these two species were considered aetiological agents because the new-borns from which they were isolated had the clinical criteria for meningitis and bacteraemia, respectively. Notably, even though these patients were bacterial and fungal culture negative, they could have had enterovirus infections; however, this issue was not examined, and this hypothesis could not be tested. Other *Bacillus* species can cause serious infections in immunosuppressed patients and are frequently associated with HCAIs in neonatal intensive care units. *Agrococcus lahaulensis* is an actinobacterium belonging to the family *Microbacteriaceae*. Certain actinobacteria are currently classified as hazardous biological agents in the workplace because actinobacteria have been increasingly detected in workplaces such as composting facilities, agricultural settings, waste management facilities, libraries and museums (Coenye et al. 2003).

These issues emphasise the need for an interdisciplinary approach to analyses of cases of nosocomial infection to avoid sample contamination and to determine whether a microorganism should be considered an aetiological agent (Rampini et al. 2011; Cuervo et al. 2008).

*Achromobacter insolitus* is an unusual or uncommon organism found only rarely in human clinical samples. The type strain LMG 6003T was isolated from a leg wound. To our knowledge, it is the first case of an HAI caused by this bacterium that has been reported in Mexico. However, because the pathogenic potential of *Achromobacter insolitus* has not been demonstrated, classification of this case as an HAI should be carefully considered and thoroughly investigated in the future.

*Leuconostoc* spp. are lactic acid bacteria that inhabit the skin, and they are commonly considered to be sample contaminants. Nevertheless, in patients with an underlying illness, *Leuconostoc* spp. could cause bacteraemia, and the number of reports of bacteraemia caused by this microorganism associated with the use of intravenous catheters is increasing (Cuervo et al. 2008; Bou et al. 2008).

The RipSeq algorithm has been validated for use with samples containing up to three different species of bacteria, although four bacterial species can occasionally be identified (Kommedal et al. 2009). Therefore, this software was applied to analyse four samples (one bronchial aspirate and three blood cultures). However, the resulting chromatograms were so complex that all of the peaks could not be included without exceeding the limitation of the RipSeq algorithm. Therefore, the obtained identifications were not dependable. Previous studies have shown that competition for reagents in mixed samples represents a major challenge in the performing of broad-range PCR and DNA sequencing directly from polybacterial samples (Kommedal et al. 2009). To minimise this problem, the use of Gram type-specific broad-range primers and the amplification of Gram-positive and Gram-negative bacteria in different tubes could be an alternative approach (Kommedal et al. 2011).

Other strategies to avoid these problems include the use of cloning and high-throughput pyrosequencing or denaturing gradient gel electrophoresis (DGGE) followed by DNA sequencing of the different fragments. Unfortunately, these diagnostic methods are even more complex, costly, labour-intensive and technically challenging than broad-range PCR and direct sequencing. In addition, these methods are associated with shortcomings with respect to the detection of minor populations in samples with large differences in the relative concentrations of the different bacterial constituents. Therefore, the next step in the resolution of polymicrobial samples is using high-throughput next generation sequencing of *16S rDNA* amplicons (Hartley and Harris 2014).

The main disadvantage of molecular diagnosis is that antimicrobial susceptibility cannot be determined. However, this study has provided sufficient information for the elucidation of molecular antimicrobial susceptibility in the near future.

In this study the variable regions V1, V2 and V3 of the *16S rDNA* gene was used (≈500 bp). All the sequences could be an assign unambiguously to a genus. The species name were determine when 98–100 % similarity to more than one GenBank sequence of the same species and/or tight clustering with members of one species (Fig. 1). A second bioinformatics analysis was performed in curated EzTaxon database to in order to confirm the sequences identification, with both methods accurate results were obtain.

## Conclusions

Our findings support the importance of the use of molecular methods as a supplemental routine method for direct bacterial identification when troubleshooting clinical specimens. The results of broad-range PCR and direct sequencing should be analysed in parallel with clinical data to determine whether the identified bacterium has medical importance or is a sample contaminant.

## Additional file

10.1186/s40064-016-1949-3 Details of antibiotic therapy alone or in combination for the cases studied herein.

## Abbreviations

CoNS: coagulase-negative staphylococci
HAIs: hospital-acquired infections
PCR: polymerase chain reaction
HCAIs: Healthcare-associated infections
CMN: Centro Médico Nacional
ISSSTE: Instituto de Seguridad y Servicios Sociales de los Trabajadores del Estado
UTI: urinary tract infection
BSI: bloodstream infection
DGGE: denaturing gradient gel electrophoresis
