# Supplementary material for: Cultivation-independent approach for the direct detection of bacteria in human clinical specimens as a tool for analysing culture-negative samples: a prospective study
Source: Springerplus. 2016 Mar 15;5:332. doi: 10.1186/s40064-016-1949-3 (PMC4792836; doi:10.1186/s40064-016-1949-3)
Supplement: Supplementary file 1 — 10.1186/s40064-016-1949-3 Details of antibiotic therapy alone or in combination for the cases studied herein. [file 40064_2016_1949_MOESM1_ESM.docx]

**Table S1. Detail of antibiotic therapy alone or in combination in the cases studied herein.**

| Case number  Table 1 - Relevant issues of the broad range PCR positive samples. | Sample type | ***16S*** ***rRNA*** identification^○^ | Hospital area | HAI | Treatment previous culture | Outcome |
| --- | --- | --- | --- | --- | --- | --- |
| 002-001 | Bronchial aspirate | Mixed infection* | ICU-A | Undemonstrated bacteraemia | Ciprofloxacin, meropenem, vancomycin, fluconazole, cefotaxime, miconazole | Improvement |
| 002-005 | CSF | *Agrococcus* sp. | ICU-N | Meningitis | UD | UD |
| 002-011 | Blood culture | Mixed infection* | BU | Undemonstrated bacteraemia | Third-generation cephalosporin | Improvement |
| 002-085 | Blood culture | *Pseudomonas* sp. | Haematology | bacteraemia | Ceftazidime, amikacin, vancomycin | Death |
| 002-088 | Blood culture | Mixed infection* | Haematology | Sample contamination | Cefepime, moxifloxacin, meropenem, amikacin, amphotericin B, linezolid | Death |
| 002-095 | Blood culture | *Bacillus* sp. | Haematology | Bacteraemia | UD | UD |
| 002-114 | Blood culture | Mixed infection* | Haematology | Bacteraemia | UD | UD |
| 002-174 | Blood culture | *Leuconostoc* sp. | Haematology | Sample contamination | Linezolid, amphotericin, cilastatin, voriconazole, cefepime, amikacin, moxifloxacin, meropenem | Improvement |
| 002-028 | Catheter tip | *Staphylococcus* sp. | BU | Catheter colonisation | Ceftriaxone, moxifloxacin | Improvement |
| 002-029 | Catheter tip | *Staphylococcus* sp. | Cardiology | Catheter colonisation | Meropenem, vancomycin, linezolid, ceftriaxone, gentamicin | Improvement |
| 002-038 | Catheter tip | *Staphylococcus* sp. | Cardiology | Catheter colonisation | Fluconazole, ceftazidime, vancomycin, amikacin | Death |
| 002-039 | Catheter tip | *Kocuria* sp. | Haematology | Catheter colonisation | UD | Improvement |
| 002-167 | Catheter tip | *Enterococcus* sp. | PI | Catheter colonisation | Dicloxacillin, cefotaxime, amikacin | Transfer |
| 002-169 | Catheter tip | *Enterococcus* sp. | Haematology | Bacteraemia | Ceftriaxone, amikacin, vancomycin | UD |
| 002-023 | Urine | *Pseudomonas* sp. | ICU-A | UTI | Linezolid, miconazole | Death |
| 002-065 | Urine | *Achromobacter* sp. | Haematology | UTI | ciprofloxacin, imipenem | Death |
| 002-103 | Urine | *Pseudocitrobacter* sp. | ICU-A | Catheter colonisation | Tigecycline | Death |
| 002-104 | Urine | *Escherichia* sp. | ICU-A | UTI | Moxifloxacin | Improvement |
| 002-105 | Urine | *Enterococcus* sp. | Neurology | UTI | Trimethoprim-sulfamethoxazole, tigecycline, cefepime, imipenem, voriconazole | Transfer |
| 002-106 | Urine | *Shigella* sp. | MMF | UTI | UD | UD |
| 002-170 | Ulcer | *Salmonella* sp. | PI | Wound | UD | UD |
| 002-037 | Wound secretion | *Staphylococcus* sp. | UTIP | STI | UD | Death |
| 002-171 | Wound secretion | *Enterococcus* sp. | Rheumatology | Wound | Imipenem, cilastatin, vancomycin, clindamycin, meropenem, ceftazidime, dicloxacillin, ciprofloxacin | Transfer |

*Aetiological agents of polybacterial infections could not be identified due to low-quality sequences. CSF: cerebrospinal fluid; ICU-A: intensive care unit, adult; ICU-N: intensive care unit, newborn; BU: burn unit; PIM: paediatric internal medicine; MMF: maternal foetal medicine; ICU-P: intensive care unit, paediatric; UTIP: urinary tract infection; PI: paediatric infectology; STI: soft tissue infection; HCAI: clinical health care infection; UD: Undetermined. ^○^The species designation was determining with the closest genetic identification based on GenBank sequence analysis (Figure 1).
